# Analysis of radiotherapy impact on survival in resected stage I/II pancreatic cancer patients: a population-based study

**DOI:** 10.1186/s12885-021-08288-4

**Published:** 2021-05-17

**Authors:** Dong Han, Fei Gao, Jin Long Liu, Hao Wang, Qi Fu, Guo Wang Yang

**Affiliations:** 1 Department of Oncology & Hematology, Beijing Hospital of Traditional Chinese Medicine, Capital Medical University, No.23 Back street in the Museum of Art Rd, Dongcheng District, Beijing, China; 2 Department of Oncology, LuanPing Hospital of Traditional Chinese Medicine, No.57, Baojian Road, Xinjian Street, Luanping Town, Chengde City, HeBei Province China

**Keywords:** Radiotherapy, Pancreatic cancer, SEER database, Survival analysis, Overall mortality

## Abstract

**Background:**

The application of radiotherapy (RT) in pancreatic cancer remains controversial.

**Aim:**

The aim of the study was to evaluate the efficacy of radiotherapy (neoadjuvant and adjuvant radiotherapy) for resectable I/II pancreatic cancer.

**Methods:**

Fourteen thousand nine hundred seventy-seven patients with pancreatic cancer were identified from SEER database from 2004 to 2015. Multivariate analyses were performed to determine factors including RT on overall survival. Overall survival and overall mortality among the different groups were evaluated using the Kaplan-Meier method and Gray’s test.

**Results:**

Patients were divided into groups according to whether they received radiotherapy or not. The median survival time of all 14,977 patients without RT was 20 months, neoadjuvant RT was 24 months and adjuvant RT was 23 months (*p* < 0.0001). Median survival time of 2089 stage I patients without RT was 56 months, significantly longer than those with RT regardless of neoadjuvant or adjuvant RT (no RT: 56 months vs adjuvant RT: 37 months vs neoadjuvant RT: 27 months, *P* = 0.0039). Median survival time of 12,888 stage II patients with neoadjuvant RT was 24 months, adjuvant RT 22 months, significantly prolonged than those without radiotherapy (neoadjuvant RT: 24 months vs adjuvant RT: 22 months vs no RT: 17 months, *P*<0.0001). Neoadjuvant RT (HR = 1.434, *P* = 0.023, 95% CI: 1.051–1.957) was independent risk factors for prognosis of stage I patients, and adjuvant RT (HR = 0.904, *P* < 0.001, 95% CI: 0.861–0.950) predicted better outcomes for prognosis of stage II patients by multivariate analysis. The risk of cancer-related death caused by neoadjuvant RT in stage I and no-RT in stage II patients were significantly higher.

**Conclusions:**

The study identified a significant survival advantage for the use of adjuvant RT over surgery alone or neoadjuvant RT in treating stage II pancreatic cancer. RT was not associated with survival benifit in stage I patients.

## Introduction

Pancreatic cancer (PC) is an extremely malignant tumor with poor outcomes. The 5-year survival is as low as 2–9% [1–3]. The incidence rate of pancreatic cancer is increasing year by year [4], and it has been estimated to rise from the fourth cause of cancer-related deaths to the second major cause of cancer-related death in the United States [5].

Surgery remains the only curative treatment for PC. However, patients with PC usually present late period and only 20% of them have a chance of undergoing surgery when diagnosed [6]. Even in resectable patients who received surgery treatment, the prognosis was not very satisfactory. Adjuvant treatment is recommended in resected pancreatic cancer with PT1–4/N0-1M0 who undergo an R0/R1 resection to reduce the recurrence rate. In the past decades, owing to the chemotherapy and radiotherapy (RT) technology development, for patients who can successfully receive surgical resection, the 5-year survival rate after adjuvant treatment accounts for 27% [7].

Chemoradiation technology has been used for resectable PC. In theory, the goal of preoperative chemoradiotherapy for PC is to purify vascular boundary, increase the possibility of negative resection at the margin and prolong survival of early treatment of micrometastatic disease. Postoperative radiotherapy can provide sufficient local control to prevent or delay the local lesions progression, and postoperative chemotherapy can reduce the recurrence rate. However, in fact, the effect of radiotherapy on the prognosis of resectable PC is controversial.

Results from ESPAC-1 trial revealed that postoperative chemoradiotherapy may not be necessary or even harmful [8]. But the conclusion is controversial due to the lack of quality control of radiation treatment. In an open-label, multicenter, randomized phase III trial [9], 132 resected patients with R0/R1 received either chemoradiotherapy group or chemotherapy group, median survival time were 26.5 and 28.5 months, respectively(*P* > 0.05). The results indicated chemoradiotherapy did not improve the survival compared with chemotherapy. A meta-analysis [10] included five randomised controlled trials of adjuvant treatment in pancreatic adenocarcinoma 939 patients, and the results showed chemoradiation is not effective adjuvant treatment in pancreatic cancer.

In contrast, other studies obtained different conclusions about the effect of radiotherapy. In the GERCOR phase II study [11], 90 patients after R0 resection of pancreatic head cancer were randomly divided into four gemcitabine treatment cycles or two gemcitabine cycles followed by gemcitabine with concurrent radiation weekly(50.4Gy/28f), the results showed median disease-free survival (DFS) was 12 months in the adjuvant chemoradiotherapy group and 11 months in the chemotherapy alone group. First local recurrence was notably lower in the chemoradiotherapy group (11% v 24%).

The contradiction of the conclusions from different studies leads to the lack of clear guidelines for the application of radiotherapy in pancreatic cancer. This retrospective study was based on a large-scale population database to evaluate the efficacy of radiotherapy (neoadjuvant and adjuvant radiotherapy) for resectable I/II PC.

## Methods

SEER (Surveillance, Epidemiology, and End Results) database of the National Cancer Institute is an important resource for population-based oncology research, documenting the information of cancer patients in some states in the United States for 40 years, with information on the diagnosis, treatment and survival data of millions of confirmed cancer patients. The number of SEER registration stations has now been expanded to eighteen.

The study extracted data using SEER*stat 8.3.6 software. Permission to access the custom data file in the SEER program was obtained and the reference number was 10,016-Nov2019. All study variables were obtained directly from the SEER database. This study was approved by Ethics Committee of Beijing Hospital of Traditional Chinese Medicine, Capital Medical University.

**Fig. 1:**
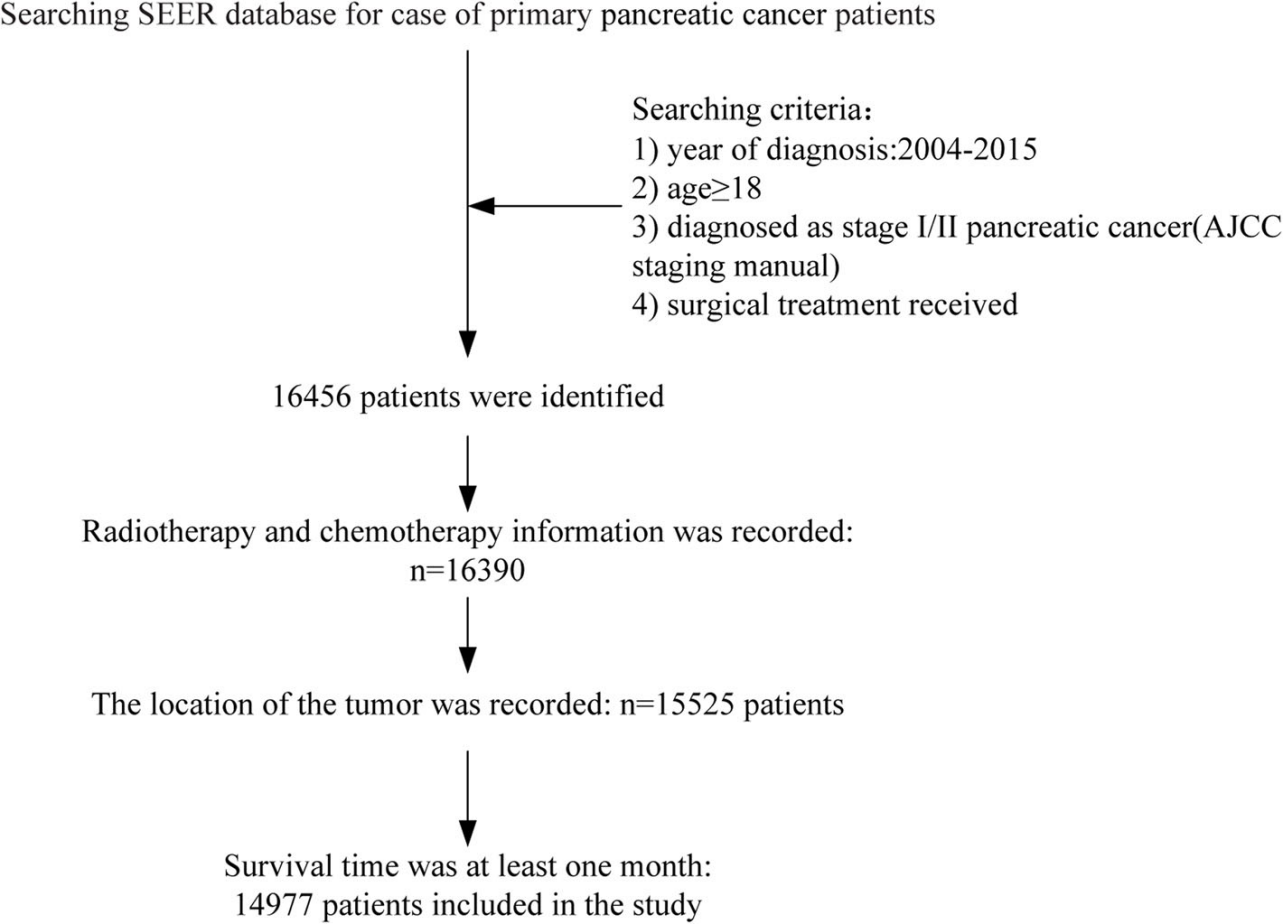
Flow chart showed selection of PC patients registered in SEER from 2004 to 2015 in this study

We extracted pancreatic cancer patients’ records data registered in SEER database from 2004 to 2015 and patients who accord with the following criteria were included in the study: (1) Adults aged ≥18; (2) Pathologically confirmed malignant tumor originated from pancreatic duct epithelium; (3) Patients who diagnosed as stage I/II pancreatic cancer according to the American Joint Committe on cancer (AJCC) staging manual (Stage I—T1: localized within the pancreas, with maximum diameter ≤ 2 cm or T2: localized within the pancreas, with a maximum diameter > 2 cm; N0: no regional lymph node metastasis; M0: no distant metastasis. Stage II—T3: extends beyond the pancreas but not involving the celiac axis or superior mesenteric artery; N0 and M0 or T1-T3/N1: regional lymph node metastasis and M0); (4) patients who received surgical resection; (5) Complete radiotherapy information record (including neoadjuvant RT, adjuvant RT and non-RT). (6) Survival time ≥ 1 month. Figure 1 displayed the cohort identification process.

The primary outcome of the study was overall survival. Overall survival was determined from the beginning of the diagnosis until death of any cause or until the last follow-up date. Secondary endpoints were cancer-related mortality. Variables extracted from the SEER database included the following: age at diagnosis, year of diagnosis, sex, race recode, primary site, ICD-O-3 Hist/behave, pathological grade, derived AJCC T, RX Summ-Surg Prim Site, radiation sequence with surgery, chemotherapy recode, survival months, vital status recode, cause of death (COD) to site recode, Cause-specific death classification, Other cause of death classification.

All statistical calculations were carried out using SPSS 19.0 software, R (version 3.6.3) and figures were drawn by Graphpad Prism 7.0 and R. All variables have been converted to category variables for better analysis. The clinical characteristics baseline among different groups were compared by Chi-square test. The overall survival analysis was estimated by Kaplan-Meier curves and compared by the Log-Rank method. The Hazard’s ratio was determined by univariate and multivariate Cox proportional hazard model. Secondary endpoint was compared using competitive risk analysis. Competition risk analysis was conducted by Gray’s test using “cmprsk” and “survival” packages in R. All statistical tests were two-sided, and *P*-value < 0.05 was considered statistically significant.

## Results

### The correlation between clinical parameters and RT in pancreatic cancer patients

From the year 2004 until the end of the year 2015, 164,035 patients with pancreatic cancer were enrolled in SEER database. In total, there were 2089 stage I patients and 12,888 stage II patients underwent surgery operation were enrolled in this study. The median age at diagnosis was 67 years old (age ranging from 18 to 97). Among stage I patients, 88 patients received neoadjuvant RT and 388 patients received adjuvant RT. The proportion of patients receiving radiotherapy differed in year of diagnosis, sex, race, primary site, pathological grade, T stage and whether received chemotherapy.

Five hundred forty-four patients received neoadjuvant RT and 4200 patients received adjuvant RT in stage II patients. The composition ratio of radiation therapy in stage II patients differed in age, year of diagnosis, race, primary site, pathological grade, T stage, N stage, whether received chemotherapy and surgical methods (*P* < 0.05). The characteristics of all patients are presented in Table 1.

**Table 1:** The correlation between clinical parameters and RT use

Clinical parameters	Stage I				Stage II			
	No RT	Neoadjuvant RT	adjuvant RT	*P*	No RT	Neoadjuvant RT	adjuvant RT	*P*
Age				0.626				<0.001
< 60	471(29.2)	26(29.5)	123(31.7)		1822(22.4)	193(35.5)	1297(30.9)	
≥ 60	1142(70.8)	62(70.5)	265(68.3)		6322(77.6)	351(64.5)	2903(69.1)	
Year of diagnosis				<0.001				<0.001
2004–2009	737(45.7)	34(38.6)	235(60.6)		3226(39.6)	158(29.0)	2101(50.0)	
2010–2015	876(54.3)	54(61.4)	153(39.4)		4918(60.4)	386(71.0)	2099(50.0)	
Sex				0.002				0.133
Female	916(56.8)	44(50.0)	183(47.2)		4038(49.6)	278(51.1)	2012(47.9)	
Male	697(43.2)	44(50.0)	205(52.8)		4106(50.4)	266(48.9)	2188(52.1)	
Race				0.012				0.009
Black	161(10.0)	12(13.6)	58(14.9)		751(9.2)	59(10.8)	394(9.4)	
White	1282(79.5)	72(81.8)	300(77.3)		6773(83.2)	466(85.7)	3492(83.1)	
Other	170(10.5)	4(4.5)	30(7.7)		620(7.6)	19(3.5)	314(7.5)	
Primary tumor Site				<0.001				<0.001
Head	880(54.6)	65(73.9)	251(64.7)		6285(77.2)	461(84.7)	3314(78.9)	
Body	233(14.4)	11(12.5)	56(14.4)		512(6.3)	36(6.6)	270(6.4)	
tail	381(23.6)	6(6.8)	54(13.9)		904(11.1)	22(4.0)	398(9.5)	
Pancreatic duct	26(1.6)	1(1.1)	7(1.8)		100(1.2)	1(0.2)	44(1.0)	
Overlapping	93(5.8)	5(5.7)	20(5.2)		343(4.2)	24(4.4)	174(4.1)	
Pathological grade				<0.001				<0.001
I	315(19.5)	12(13.6)	57(14.7)		768(9.4)	42(7.7)	370(8.8)	
II	608(37.7)	22(25.0)	202(52.1)		3781(46.4)	166(30.5)	2094(49.9)	
III	272(16.9)	18(20.5)	89(22.9)		2878(35.3)	106(19.5)	1450(34.5)	
IV	29(1.8)	0(0)	5(1.3)		112(1.4)	8(1.5)	53(1.3)	
Unknown	389(24.1)	36(40.9)	35(9.0)		605(7.4)	222(40.8)	233(5.5)	
T				<0.001				0.002
1	636(39.4)	20(22.7)	115(29.6)		157(1.9)	2(0.4)	75(1.8)	
2	977(60.6)	68(77.3)	273(70.4)		582(7.1)	23(4.2)	325(7.7)	
3	–	–	–		7405(90.9)	519(95.4)	3800(90.5)	
N				–				<0.001
0	–	–	–		2384(29.3)	256(47.1)	1025(24.4)	
1	–	–	–		5760(70.7)	288(52.9)	3175(75.6)	
Chemotherapy				<0.001				<0.001
No	1041(64.5)	3(3.4)	28(7.2)		3656(44.9)	8(1.5)	215(5.1)	
Yes	572(35.5)	85(96.6)	360(92.8)		4488(55.1)	536(98.5)	3985(94.9)	
Surgical procedures				0.082				0.010
Local excision of tumor	12(0.7)	1(1,1)	2(0.5)		27(0.3)	5(0.9)	19(0.5)	
Pancreatectomy	1411(87.5)	67(76.1)	341(87.9)		6916(84.9)	447(82.2)	3569(85.0)	
Extended	154(9.5)	15(17.0)	34(8.8)		1097(13.5)	78(14.3)	577(13.7)	
Unknown	36(2.2)	5(5.7)	11(2.8)		104(1.3)	14(2.6)	35(0.8)	

*Abbreviations*: *RT* Radiotherapy. Note: Pathological grade I, Well differentiated; II, Moderately differentiated; III, Poorly differentiated; IV, Undifferentiated; anaplastic. Pancreatectomy: partial pancreatectomy, local or partial pancreatectomy and duodenectomy with/without distal/partial gastrectomy, total pancreatectomy; Extended: total pancreatectomy and subtotal gastrectomy or duodenectomy, extended pancreatoduodenectomy

### Overall survival analysis of stage I/II pancreatic cancer patients

**Fig. 2:**
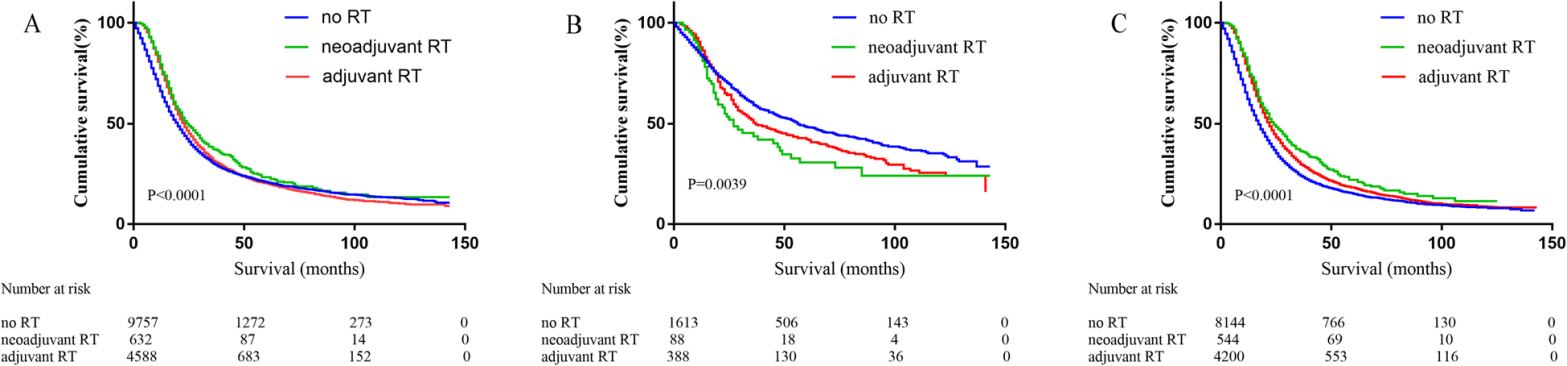
Overall survival of patients stratified by RT. **a** Overall survival of 14,977 cases of stage I/II patients treated without RT (*n* = 9757) versus patients treated with neoadjuvant RT (*n* = 632) versus patients treated with adjuvant RT (*n* = 4588) (*P* < 0.0001). **b** Plot of overall survival for 2089 stage I patients stratified by RT use. Patients not received RT (*n* = 1613) versus patients received neoadjuvant RT (*n* = 88) versus patients treated with adjuvant RT (*n* = 388) (*P* = 0.0039). **c** Plot of overall survival for 12,888 stage II patients stratified by RT use. Patients not received RT (*n* = 8144) versus patients received neoadjuvant RT (*n* = 544) versus patients treated with adjuvant RT (*n* = 4200) (*P* < 0.0001)

Fourteen thousand nine hundred seventy-seven patients with pancreatic cancer were divided into three groups according to whether they received RT or not. Kaplan-Meier (KM) analysis showed that the median survival time of patients without RT was 20 months, neoadjuvant RT was 24 months and adjuvant RT was 23 months (*p* < 0.0001, Fig. 2a). We performed subgroup analysis according to different stages.

Two thousand eighty-nine cases of stage I pancreatic cancer patients were divided into three groups by radiotherapy. Comparison of the median survival time differences was conducted in the three groups. Median survival time of patients without RT was 56 months, significantly longer than the median survival time of those with RT regardless of neoadjuvant or adjuvant RT (no RT: 56 months vs adjuvant RT: 37 months vs neoadjuvant RT: 27 months, *P* = 0.0039, Fig. 2b). Similarly, all 12,888 patients with stage II pancreatic cancer were divided into three groups according to whether they received radiotherapy or not. Median survival time of patients with neoadjuvant RT was 24 months, adjuvant RT 22 months, significantly prolonged than the median survival time of those without radiotherapy (neoadjuvant RT: 24 months vs adjuvant RT: 22 months vs no RT: 17 months, P<0.0001, Fig. 2c).

**Fig. 3:**
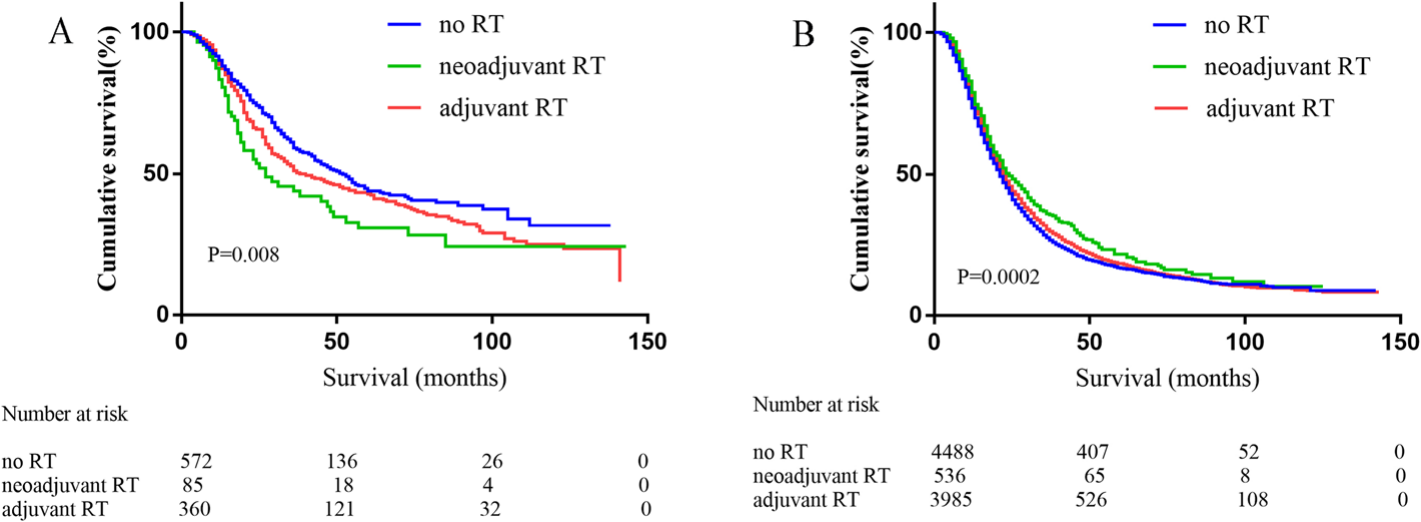
Overall survival of chemotherapy patients stratified by RT. **a** Plot of overall survival for 1017 stage I patients received chemotherapy stratified by RT use. Patients not received RT (*n* = 572) versus patients received neoadjuvant RT (*n* = 85) versus patients treated with adjuvant RT (*n* = 360) (*P* = 0.008). **b** Plot of overall survival for 9009 stage II patients stratified by RT use. Patients not received RT (*n* = 4488) versus patients received neoadjuvant RT (*n* = 536) versus patients treated with adjuvant RT (*n* = 3985) (*P* = 0.0002)

In addition, only a small number of surgically treated patients received radiotherapy alone, the number of patients who underwent radiotherapy alone for neoadjuvant and adjuvant therapy were only 11 and 243 patients, respectively. Therefore, we also analyzed whether the addition of radiotherapy can improve the survival of surgically treated patients in the chemotherapy group. Among stage I patients, 1017 patients received chemotherapy. The results showed that the addition of radiotherapy did not improve the prognosis of patients (neoadjuvant RT: 27 months vs adjuvant RT: 40 months vs no RT: 52 months, *P* = 0.008, Fig. 3a). Nine thousand nine cases received chemotherapy in stage II patients, the results showed median survival time was 24 months in neoadjuvant RT group, 22 months in adjuvant RT group and 21 months in chemotherapy alone group(*P* = 0.0002, Fig. 3b). The median survival time of patients receiving RT (neoadjuvant RT or advanced RT) was longer than those without RT.

### Univariate analysis of clinical features affecting the prognosis

Univariate survival analysis was separately performed for stage I/II patients. For stage I patients, the unadjusted protective factor was tumor located in tail of pancreas. Radiotherapy including age (≥ 60 years old), male, pathology with worse differentiation, higher T stage were adverse diagnostic factors.

For patients with stage II pancreatic cancer, radiotherapy, chemotherapy and newly diagnosed pancreatic cancer after 2010 were the advantageous factors for the prognosis of patients. The harmful factors for the prognosis included age (≥ 60 years old), male, pathology with worse differentiation, higher T and higher N stage. The results are shown in Table 2.

**Table 2:** Univariate analysis of clinical features affecting the prognosis of pancreatic cancer patients

Clinical parameters	Stage I			Stage II		
	HR	95%CI	*P*	HR	95%CI	*P*
Age						
< 60	Ref			Ref		
≥ 60	2.063	1.772–2.401	<0.001	1.263	1.204–1.324	<0.001
Year of diagnosis						
2004–2009	Ref			Ref		
2010–2015	0.897	0.785–1.024	0.109	0.866	0.830–0.902	<0.001
Sex						
Female	Ref			Ref		
Male	1.279	1.131–1.446	<0.001	1.042	1.000–1.085	0.049
Race						
Black	Ref			Ref		
White	1.077	0.880–1.319	0.470	0.959	0.895–1.028	0.242
Other	0.760	0.567–1.020	0.067	0.911	0.823–1.008	0.072
Primary tumor Site						
Head	Ref			Ref		
Body	0.831	0.690–1.002	0.052	0.975	0.894–1.063	0.568
tail	0.606	0.512–0.718	<0.001	0.909	0.848–0.974	0.007
Pancreatic duct	0.856	0.536–1.368	0.517	0.876	0.725–1.058	0.169
Overlapping	0.773	0.583–1.025	0.074	1.072	0.968–1.188	0.179
Pathological grade						
I	Ref			Ref		
II	1.811	1.499–2.187	<0.001	1.440	1.331–1.559	<0.001
III	2.570	2.085–3.168	<0.001	1.902	1.754–2.061	<0.001
IV	1.746	1.056–2.889	0.030	1.684	1.394–2.033	<0.001
Unknown	1.039	0.829–1.303	0.739	1.147	1.031–1.276	0.012
T						
1	Ref			Ref		
2	1.371	1.201–1.564	<0.001	1.315	1.113–1.554	0.001
3	–			1.295	1.113–1.506	0.001
N						
0	–			Ref		
1	–			1.446	1.380–1.516	<0.001
Chemotherapy						
No	Ref			Ref		
Yes	1.056	0.933–1.194	0.389	0.637	0.610–0.666	<0.001
Surgical procedures						
Local excision of tumor	Ref			Ref		
Pancreatectomy	0.900	0.428–1.894	0.782	0.752	0.551–1.026	0.072
Extended	0.962	0.447–2.071	0.922	0.820	0.598–1.123	0.215
Unknown	1.212	0.529–2.775	0.650	0.791	0.550–1.135	0.203
Radiotherapy						
No		Ref		Ref		
neoadjuvant RT	1.468	1.099–1.960	0.009	0.690	0.617–0.770	<0.001
adjuvant RT	1.193	1.028–1.384	0.020	0.790	0.756–0.825	<0.001

*Abbreviations*: *RT* Radiotherapy. Note: Pathological grade I, Well differentiated; II, Moderately differentiated; III, Poorly differentiated; IV, Undifferentiated; anaplastic. Pancreatectomy: partial pancreatectomy, local or partial pancreatectomy and duodenectomy with/without distal/partial gastrectomy, total pancreatectomy; Extended: total pancreatectomy and subtotal gastrectomy or duodenectomy, extended pancreatoduodenectomy

### Multivariate analysis of clinical features affecting the prognosis

Multivariate survival analysis of stage I pancreatic cancer showed that neoadjuvant RT (HR = 1.434, *P* = 0.023, 95% CI: 1.051–1.957), age (≥60 years old), male, pathology with worse differentiation, higher T stage were independent risk factors for prognosis, indicating a shorter survival period. Chemotherapy, tumor located in body and tail of pancreas, non-white/black Americans were favorable prognostic factors and related to longer survival period.

Also, we conducted multivariate survival analysis for stage II pancreatic cancer patients. Results showed that adjuvant RT (HR = 0.904, *P* < 0.001, 95% CI: 0.861–0.950) including chemotherapy, expanded surgical procedure, pancreatic duct tumors, diagnosed pancreatic cancer after 2010 and non-black Americans predicted better outcomes. On the contrary, age (≥60 years old), male, pathology with worse differentiation, higher T and N stage predicted worse outcomes, which means shorter survival time. Multivariate analysis results are shown in Table 3.

**Table 3:** Multivariate analysis of clinical features affecting the prognosis of pancreatic cancer patients

Clinical parameters	Stage I			Stage II		
	HR	95%CI	*P*	HR	95%CI	*P*
Age						
< 60	Ref			Ref		
≥ 60	1.894	1.621–2.213	<0.001	1.220	1.162–1.280	<0.001
Year of diagnosis						
2004–2009	Ref			Ref		
2010–2015	0.943	0.823–1.080	0.394	0.887	0.850–0.926	<0.001
Sex						
Female	Ref			Ref		
Male	1.172	1.035–1.327	0.012	1.055	1.013–1.100	0.010
Race						
Black	Ref			Ref		
White	0.971	0.790–1.192	0.776	0.907	0.846–0.973	0.006
Other	0.705	0.524–0.949	0.021	0.889	0.802–0.985	0.024
Primary tumor Site						
Head	Ref			Ref		
Body	0.820	0.679–0.989	0.038	1.049	0.961–1.145	0.281
tail	0.698	0.587–0.830	<0.001	0.953	0.888–1.023	0.181
Pancreatic duct	1.150	0.717–1.845	0.563	0.789	0.653–0.954	0.014
Overlapping	0.772	0.581–1.027	0.076	1.088	0.982–1.205	0.107
Pathological grade						
I	Ref			Ref		
II	1.719	1.418–2.084	<0.001	1.466	1.354–1.587	<0.001
III	2.318	1.867–2.878	<0.001	1.947	1.796–2.112	<0.001
IV	1.861	1.121–3.089	0.016	1.807	1.495–2.183	<0.001
Unknown	1.010	0.803–1.268	0.935	1.273	1.142–1.419	<0.001
T						
1	Ref			Ref		
2	1.412	1.234–1.615	0.000	1.274	1.078–1.506	0.004
3	–			1.450	1.245–1.690	<0.001
N						
0	–			Ref		
1	–			1.543	1.469–1.621	<0.001
Chemotherapy						
No	Ref			Ref		
Yes	0.827	0.727–0.940	0.004	0.628	0.598–0.660	<0.001
Surgical procedures						
Local excision of tumor	Ref			Ref		
Pancreatectomy	0.677	0.320–1.433	0.308	0.647	0.474–0.884	0.006
Extended	0.642	0.296–1.393	0.263	0.702	0.512–0.963	0.028
Unknown	1.208	0.524–2.784	0.657	0.713	0.496–1.025	0.068
Radiotherapy						
No	Ref			Ref		
neoadjuvant RT	1.434	1.051–1.957	0.023	0.975	0.868–1.095	0.668
adjuvant RT	1.095	0.918–1.307	0.314	0.904	0.861–0.950	<0.001

*Abbreviations*: *RT* Radiotherapy. Note: Pathological grade I, Well differentiated; II, Moderately differentiated; III, Poorly differentiated; IV, Undifferentiated; anaplastic. Pancreatectomy: partial pancreatectomy, local or partial pancreatectomy and duodenectomy with/without distal/partial gastrectomy, total pancreatectomy; Extended: total pancreatectomy and subtotal gastrectomy or duodenectomy, extended pancreatoduodenectomy

### Competitive risk analysis for cancer-related death of I/II pancreatic cancer patients

**Fig. 4:**
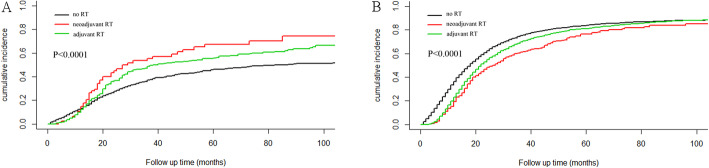
Competitive risk analysis for cancer-related death of patients stratified by RT. **a** Cancer-related death of stage I patients caused by RT use or not. (*P* < 0.0001). **b** Cancer-related death of patients stage II patients caused by RT. (*P* < 0.0001)

The causes of death were divided into cancer-related death and non-cancer-related death. We analyzed the death outcomes by competitive risk model. The results showed that the 1-, 3-, and 5-year cancer-related mortality rate in stage I patients treated with neoadjuvant RT were 13.98%, 52.33 and 63.58% respectively. In adjuvant RT group, the 1-year, 3-year and 5-year cancer-related mortality rates were 10.95, 43.16 and 48.94%, nevertheless these rates decreased to 11.54, 32.02 and 39.51% respectively in patients without radiotherapy. The risk of cancer-related death caused by neoadjuvant RT was significantly higher than adjuvant RT and no-RT (*p* < 0.0001; Fig. 4a) .

The 1-year, 3-year and 5-year cancer-related mortality rates in patients with stage II pancreatic cancer without radiotherapy were 32.70, 66.78 and 74.16% respectively, In patients who received adjuvant RT, the 1-year, 3-year and 5-year cancer-related mortality rates were 21.16%, 63.47 and 73.38%. The rates were 17.57%, 55.30 and 68.49% in neoadjuvant RT group. The results showed the cancer-related death of II stage patients without radiotherapy was significantly higher than that of patients received radiotherapy(*P* < 0.0001; Fig. 4b).

## Discussion

Although PC in early stage can be treated surgically, the complete resection rate is low due to anatomical position of pancreas complexity, the recurrence rate can up to 80% [12]. Previous studies have confirmed compared with surgery treatment only, postoperative adjuvant chemotherapy has positive effect by preventing or delaying tumor recurrence and improving the long-term survival rate. At 2008 ASCO annual meeting, the CONKO-001 study reported by the investigator showed that adjuvant chemotherapy benefits patients’ survival after pancreatic cancer surgery. In CONKO-001 phase III clinical trial [13, 14], 354 patients with PC after resection were randomly divided into adjuvant gemcitabine (GEM) treatment group or observation group. The results showed GEM adjuvant chemotherapy improved Disease-free Survival (DFS) (13.4 months vs 6.9 months, *P* < 0.001). The long-term follow-up results showed that the total survival time of patients in GEM group was significantly superior than observation group (the mOS was 22.8 months vs 20.2 months, the 5-year survival rate was 20.7% vs 10.4%, and the 10-year survival rate was 12.2% vs 7.7%, respectively, *p* < 0.01). The study supported conclusion that adjuvant chemotherapy compared with observation alone resulted in increased overall survival. Before CONKO-001, adjuvant chemotherapy was not common for pancreatic cancer. In this study, we have included cases since 2004 for two reasons: First, in the SEER database, most of the cases after 2004 (including 2004) recorded AJCC stage, while only a small part of cases before 2004 recorded AJCC stage, our retrospective study included more cases in order to conclude more reliable conclusions. Second, among the cases we included, there were still 2304 patients who received chemotherapy between 2004 and 2007, and there may be a number of patients received postoperative adjuvant chemotherapy (neoadjuvant chemotherapy or postoperative adjuvant chemotherapy is not recorded in the SEER database). Our study also showed that chemotherapy is an independent protective factor for the prognosis of pancreatic cancer. Chemotherapy reduced the risk of death by 17.3% in patients with stage I and 37.2% in patients with stage II.

Adjuvant chemotherapy is necessary for resectable pancreatic cancer, but the role of radiotherapy remains controversial due to opposite conclusions drawn from different studies. The results of EROTC-40891 showed that there was no significant difference in survival rate and progression free survival rate between the chemoradiotherapy group and the observation group, and adjuvant chemoradiotherapy did not improve survival [7]. Results from ESPAC-1 trial identified adjuvant chemotherapy has significant survival benefits for patients with pancreatectomy, while adjuvant chemoradiotherapy has adverse effects on survival [8, 10, 15]. But other studies held the opposite conclusion. Since a randomized, prospective, multi-institutional Gastrointestinal Tumor Study Group (GITSG) trial [16, 17] had supported postoperative adjuvant radiotherapy and chemotherapy, subsequent clinical trials also demonstrated a fact chemoradiotherapy had survival advantage for resected patients. Results from a prospective database of 616 pancreatic cancer patients after resection of pancreatic cancer at Johns Hopkins Hospital suggested adjuvant chemoradiotherapy significantly improves survival when compared with patients observation alone [18]. The Mayo Clinic conducted a retrospective review of 466 patients who underwent R0 resection of pancreatic cancer, and found that overall survival was better in patients who received adjuvant chemoradiotherapy than those not received adjuvant chemoradiotherapy. Also, there were two large-scale population-based retrospective studies supported radiotherapy as an adjuvant treatment for localized pancreatic cancer. In 2008, Stessin et al. [19] analyzed 3885 cases of surgically resected stage I/II pancreatic cancer patients in SEER database from 1994 to 2003. Overall survival of patients receiving neoadjuvant RT was 23 months, while patients without RT was 12 months, and patients receiving adjuvant RT was 17 months. The analysis showed a survival benefit for the use of neoadjuvant RT in treating pancreatic cancer. In 2010, McDade et al. [20] analyzed 5676 cases of resected pancreatic adenocarcinoma in SEER database from 1988 to 2005. The median survival time (18 months) of patients with adjuvant radiotherapy was better than those without adjuvant radiotherapy (10 months, *P* < 0.0001). These studies have come to different conclusions presumably because of the patients included with different baseline characteristics, staging, treatment methods and doses.

Neoadjuvant radiotherapy alone was rarely used in patients with resectable pancreatic cancer. First, pancreatic cancer represents highly resistant to radiation, the response rate to radiotherapy was low. Second, the pancreas is a retroperitoneal organ with a deep anatomic position, surrounded by important organs such as stomach, liver and other organs. The effect of radiotherapy is affected by many factors such as target area delineation, peristalsis of surrounding organs. Another barrier is the intolerance of surrounding organs to high dose radiotherapy, which leads to the radiation effect limitation. Furthermore, the use of neoadjuvant radiotherapy for resectable pancreatic cancer may delay surgery, providing an opportunity for tumor metastasis that becomes unresectable. These barriers limited the application of neoadjuvant radiotherapy in pancreatic cancer. Our results displayed in the resectable pancreatic cancer, the number of patients receiving neoadjuvant radiotherapy was small, only 88 (4.21%) in stage I and 544 (4.22%) in stage II patients.

Different from the studies of Stessin and McDade, we performed a subgroup analysis of resectable pancreatic cancer according to TNM staging. Dramatic differences are observed in radiotherapy effect on stage I and II pancreatic cancer patients. Our study showed that for stage I pancreatic cancer patients, KM analysis indicated the median survival time of patients with no radiotherapy was significantly longer than those with radiotherapy, and the median survival time of patients received neoadjuvant radiotherapy is the shortest (56 months with no RT vs 37 months with adjuvant RT vs 27 months with neoadjuvant RT, *P* = 0.0039, Fig. 2b). Even for patients received chemotherapy, the addition of radiotherapy use shortened survival time (Fig. 3a). Multivariate Cox analysis showed that neoadjuvant radiotherapy was an independent risk factor. The 1-, 3-and 5-year risk of cancer-related death of neoadjuvant radiotherapy was significantly higher than non-radiotherapy and adjuvant radiotherapy. Similar to the previous study, Hazard et al. [21] analyzed 3008 pancreatic patients who underwent resection without distant metastasis from 1988 to 2002 in SEER database. Multivariate analysis showed that radiation therapy played a positive role in overall survival for patients who had direct extension beyond the pancreas and/or regional lymph node involvement (*P* < 0.01) but not for patients with T1-T2N0M0 disease (*P* > 0.05). It was necessary to unify the conditions for chemotherapy if the effects of radiotherapy were to be compared. While in SEER database, records of factors such as chemotherapy cycles, regimen and dose are not listed, only whether chemotherapy received or not was recorded. Therefore, due to the limitations of the records, we did not unify the conditions of chemotherapy. It is noteworthy that in our study, Among stage I patients received neoadjuvant radiotherapy, of whom 15 cases underwent total pancreatectomy and subtotal gastrectomy or duodenectomy/extended pancreatoduodenectomy. We speculated these 15 patients may have high-risk factors that require neoadjuvant chemotherapy to improve R0 resection rate, the characteristics of these patients may mean a worse prognosis, leading to the conclusion that neoadjuvant radiotherapy has a negative effect in stage I patients.

For patients with stage II pancreatic cancer, the effect of radiotherapy on survival was completely opposite. KM analysis indicated that radiotherapy prolonged the patients’ survival time, and the median survival of patients without radiotherapy is the shortest (17 months with no RT vs 22 months with adjuvant RT vs 24 months with neoadjuvant RT, *P* < 0.0001, Fig. 2c), similar results were obtained in the chemotherapy group (Fig. 3b). Cox analysis showed that adjuvant radiotherapy is an independent protective factor. Cancer-related death risk of no radiotherapy in 1-, 3-and 5-year was significantly higher than neoadjuvant and adjuvant radiotherapy. Our conclusion was consistent with Moody’s study. Moody et al. [22] analyzed the record of 3252 patients with pancreatic cancer after operation in SEER database, the results showed that radiotherapy could significantly improve the survival time of patients with stage II B (T1-T3N1). Because the sequence of chemotherapy was not recorded in SEER database, we had to analyze radiotherapy and chemotherapy separately. Our results demonstrated that the addition of adjuvant radiotherapy could prolong the survival period and reduce tumor related death for stage II pancreatic cancer.

## Conclusion

By the analysis of resectable stage I/II pancreatic cancer subgroups, our results demonstrated a survival benefit for adjuvant RT use in resected stage II pancreatic cancer. RT especially neoadjuvant RT may be associated with a worse survival in stage I patients. Our study was based on a large national cancer registration database to provide more evidence for the application of radiotherapy in operable stage I/II pancreatic cancer patients. It is undeniable that our research was a retrospective study and bias was inevitable. We try to minimize this bias through a large data analysis and statistical method. Also, other important prognostic factors, including surgical margins, lymphovascular invasion, performance status and comorbidities of patients, and chemotherapy information (such as chemotherapy cycles, regimen and dose, whether neoadjuvant chemotherapy or adjuvant chemotherapy) did not included in the study due to unavailable in SEER database, we did not incorporate these factors into our study, which led to some limitations. So further prospective clinical trials are needed.

## Data Availability

Data files were downloaded directly from the SEER website.

## Abbreviations

SEER: Surveillance, Epidemiology, and End Results
RT: Radiotherapy
OS: Overall survival
PC: Pancreatic cancer
AJCC: American Joint Committe on cancer
COD: Cause of death
KM: Kaplan-Meier
DFS: Disease-free Survival
GEM: Gemcitabine
GITSG: Gastrointestinal Tumor Study Group
